# Conditioned medium of primary lung cancer cells induces EMT in A549 lung cancer cell line by TGF-ß1 and miRNA21 cooperation

**DOI:** 10.1371/journal.pone.0219597

**Published:** 2019-07-25

**Authors:** Rosa Camerlingo, Roberta Miceli, Laura Marra, Giuseppina Rea, Igea D’Agnano, Marta Nardella, Roberta Montella, Alessandro Morabito, Nicola Normanno, Virginia Tirino, Gaetano Rocco

**Affiliations:** 1 SC Cell Biology and Biotherapy, Istituto Nazionale Tumori IRCCS, Fondazione G. Pascale, Naples, Italy; 2 reiThera srl, Rome, Italy; 3 Molecular Immunology and Immunoregulation, Istituto Nazionale Tumori IRCCS, Fondazione G. Pascale, Naples, Italy; 4 Institute of Cell Biology and Neurobiology-CNR, Monterotondo, Rome, Italy; 5 Institute for Biomedical Technologies-CNR, Segrate, Milan, Italy; 6 Department of Neurosciences, Unit of Neuromuscular and Neurodegenerative Disorders, Bambino Gesù Children's Hospital, IRCCS, Rome, Italy; 7 Department of Experimental Medicine, Section of Biotechnology, Medical Histology and Molecular Biology, University of Campania “L. Vanvitelli”, Naples, Italy; 8 Thoracic Medical Oncology, Istituto Nazionale Tumori IRCCS, Fondazione G. Pascale Naples, Italy; 9 Thoracic Service, Department of Surgery, Memorial Sloan-Kettering Cancer Center, NY, United States of America; Seoul National University College of Pharmacy, REPUBLIC OF KOREA

## Abstract

The epithelial-mesenchymal transition (EMT) plays a key role in tumor progression, drug resistance and metastasis. Recently, numerous microRNA (miRNA) have been described to regulate EMT in tumor progression. In this study, we found that conditioned medium from the LC212 non-small-cell lung cancer (NSCLC) cell line (LC212-CM) induces morphological changes and overexpression of Vimentin, CD90, SMAD 2/3, SLUG and TWIST in A549 NSCLC cells, consistent with a mesenchymal phenotype. To identify the soluble mediators in LC212-CM involved in this phenomenon, we performed miRNA profiling and TGF-β1 quantification. We found that LC212-CM contains high levels of TGF-β1 as well as different secreted miRNAs. We focused our attention on Homo sapiens-microRNA21 (hsa-miR21), one of most relevant miRNA associated with lung cancer progression, metastasis and EMT. An hsa-miR21 antagomiR was able to prevent the LC212-CM-induced EMT phenotype in A549 cells. Furthermore, we found that TGF-β1 and hsa-miR21 cooperate in the induction of EMT in A549 cells. Intriguingly, TGF-β1 was found to induce hsa-miR21 expression in A549 cell, thus suggesting that the hsa-miR21 mediates at least in part the pro-EMT effects of TGF-β1. In conclusion, hsa-miR21 and TGF-β1 are involved in autocrine and paracrine circuits that regulate the EMT status of lung cancer cells.

## Introduction

Lung cancer is the leading cause of cancer related death worldwide [1]. Non small cell lung cancer (NSCLC) is the most frequent type of lung cancer [2]. It accounts for about 80% of cases and is associated to a 5-year overall survival rate of less than 15% [3,4]. The occurrence of metastasis in lung cancer patients is associated with poor prognosis. Although the surgical and chemo/radiotherapy treatments have improved over the years, the incidence of mortality for NSCLC patients remains high [3].

Recent evidence suggests that the epithelial-mesenchymal transition (EMT) promotes tumor cell migration, invasion and metastasis [5,6]. The EMT is a process through which tumor cells undergo a morphological switch from the epithelial polarized phenotype to the mesenchymal fibroblastoid phenotype. During EMT, there is a downregulation of epithelial differentiation markers, including cytokeratins and e-cadherin, and transcriptional induction of mesenchymal markers such as vimentin, fibronectin and n-cadherin with a nuclear localization of β-catenin [7,8]. Moreover, several studies have demonstrated that EMT is correlated with cancer stem cells (CSCs) phenotype [9–12]. In this context, our group has demonstrated that the induction of EMT by TGF-β1 in primary lung cancer cells results in the acquisition of a mesenchymal profile and expression of stem cell markers [9,13].

In recent years, numerous studies have reported that altered miRNA expression may be associated with cancer development and metastasis as tumor suppressors or oncogenes [14,15].

MiRNAs are a class of small, non-coding RNAs (21–24 nt in length) which are encoded by genomes in higher eukaryotes and post-transcriptionally regulate gene expression. They are able to control several biological processes including cell growth, proliferation, differentiation and apoptosis [16,17].

A large number of miRNAs has already been described as potential diagnostic and therapeutic targets for cancer [14]. In particular, Homo sapiens-microRNA21 (hsa-miR21) has been described as a potential serum and prognostic biomarker in NSCLC [18]. Nevertheless, the molecular mechanism underlying the role of hsa-miR21 in the pathogenesis and progression of lung cancer remains to be clarified.[19–21]

In our laboratory, we isolated a primary lung cancer cell line, whose culture medium was able to induce EMT in NSCLC cell lines. Therefore, the aim of this study was to investigate the effect of the conditioned medium (CM) derived from the primary LC212 lung cell line on A549 NSCLC cells and identify the potential molecular mechanism by which EMT was induced.

## Materials and methods

### Establishment of a primary LC212 cell culture, and cell culture of A549

A biopsy of lung adenocarcinoma (LC212) was obtained from a consenting male patient (57 years) undergoing lung resection in October 2011 at the Division of Thoracic Surgery of the National Cancer Institute of Naples. The diagnosis was based on clinical and histological criteria. Tumor specimen was minced with scissor and then digested by incubation over night at 37°C in RPMI 1640 containing I Type collagenase, 1mg/ml and dispase 1mg/ml (all purchased from Sigma Chemical Co., St. Louis, MO). After digestion, the cell suspension was filtered through a 70 nm nylon meshes. The cells were cultured in three different media to allow the adhesion and growth of tumor cells: (i) RPMI plus 10% FBS, 2 mM l-glutamine, 100 U/ml penicillin, 100 g/ml streptomycin (all purchased from Invitrogen, San Giuliano Milanese, Milan, Italy); (ii) Bronchial Epithelial Cell Basal Medium (BEBM) supplemented with BEGM (prepackaged SingleQuots containing retinoic acid, bovine pituitary extract, insulin, hydrocortisone, transferrin, triiodothyronine, epinephrine, human epidermal growth factor, gentamicin, and amphotericin B) [all from Lonza Group Ltd., Basel, Switzerland] and (iii) RPMI/BEBM at the mixture of 3:1, respectively. Then, the cells were cultured in a humidified incubator at 37°C under 5% CO2 atmosphere. In order to evaluate cell growth curves, LC212 cells were cultured in RPMI-1640 for 24h, 48h, 72h, 96h 120h,144h at a density of 50.000 cells/well in p6/W. A549 cell line was purchased from ATCC Cell Bank and was cultured in RPMI 1640 plus 10% FBS, 2 mM l-glutamine, 100 U/ml penicillin, 100 g/ml streptomycin (all purchased from Invitrogen, San Giuliano Milanese, Milan, Italy) at 37°C, 5% CO2. For experiments, cells were grown to 90% confluence.

### LC212 phenotype by flow cytometry and immunofluorescence

To evaluate the phenotype of LC212 cell line, it was examined the expression of the following markers by flow cytometry at day of surgical, after five (5P) and after fifteen (15P) passages of culture. We evaluated the expression of: mouse anti-human CD90 FITC (clone DG3, Miltenyi Biotech) and mouse anti-human CD90 PE-Cy5 (clone 5-E10, BD biosciences), mouse anti-human-CD133 PE (clone 293C3, Miltenyi Biotech), mouse anti-human CD326 PE (EpCAM), (clone HEA-125, Miltenyi Biotech) and mouse anti-human CD45 Vioblue (clone 5B1, Miltenyi Biotech). The antibodies (2ug/ml) were incubated for 30 minutes at 4°C in the dark. After incubation, the samples were washed in PBS and analyzed by FACS ARIA III (Becton Dickinson). All data were analyzed by Diva 8 Software. For immunofluorescence assay, LC212 cell line was analyzed at 15P of culture. It was analyzed the expression of mouse anti-human e-cadherin (clone NCH-38, DAKO), mouse anti-human cytokeratin (clone AE1/AE3, DAKO), (epithelial markers), and mouse anti-human Vimentin (cone V9, DAKO) (mesenchymal marker). All primary antibodies were used 1:500 in PBS. For immunofluorescence staining, LC212 cells were plated in 24 well plates and were fixed with 70% ethanol, 0,1% triton for 30 minutes at 4°C, washed with PBS, treated with 5% Bovine Serum Albumin for 60 minutes at room temperature and then stained with primary antibodies at 4°C over night. The secondary antibody, goat anti-mouse FITC (AbCAM) diluted 1:200 in PBS, was incubated for 60 min at 4°C, and the DAPI (Sigma, Milan, Italy), used to stain the nucleus, was incubated for 7 minutes at room temperature. Cells were then washed twice as described above and observed under the fluorescence microscope (Zeiss, Milan, Italy). Isotypes and non probed cells were used as controls.

### LC212 and LC31 conditioned medium collection and cell culture

To prepare conditioned media (CM), LC212 and LC31 cells [22] were cultured in standard medium at a density of 200.000 cells/fl 25. After 24 h the cells were washed and cultured with RPMI deprived of FBS for 48 h. To remove cells and cell debris, the collected media were centrifuged for 10 min at 14,000 rpm and 4°C, and supernatants were used as conditioned media study. A549 cells were cultured at a density of 75.000 cells/well in six wells. After 48h of culture in the standard medium, A549 cells were cultured for 96 hours with LC212 and LC31-CM, and with standard medium alone. Cell morphology was captured using an inverted microscope (Axiovert 10 ZEISS).

### Real-Time PCR

Total RNA was extracted using TRIzol Reagent (Invitrogen, Milan, Italy) in according to the manufacturer’s protocol. RNA concentration and purity were determined by A260 and A260/A280 ratios, respectively. The integrity of total RNA was assessed on standard 1% agarose/formaldehyde gels. The RNA samples were treated with DNase I to remove residual traces of DNA. Expression levels of Slug and Twist were performed by Real-Time PCR. All reactions were performed using StepOne Thermocycler (Applied Biosystems, Monza, Italy). Three reaction mixture for the amplification consisted of SYBR Green PCR Master Mix, (Applied Biosystems, Monza, Italy),250nM of each primer and 50ng of cDNA. The thermal cycling conditions were: 94°C for 2 min followed by an initial denaturation step at 95°C for 2 min, 40 cycles at 95°C for 30s, 60°C for 30s and 72°C for 30s. Real-Time PCR was performed using the following primer sequences: SLUG: fw: 5’-GAGCATTTGCAGACAGGTCA-3’; rev: 3’-CCTCATTGTTTGTGCAGGAGA-5’ and TWIST: fw: 5’-TCTCGGTCTGGAGGATGGAG-3’; rev: 3’-GTTATCCAGCTCCAGAGTCT-5’

### RNase treatment

LC212 cells has been treated with 20ul RNase (1mg/ml) for 24 h. and the conditioned medium obtained was added to A549 cells. A549 cells were cultured in a P6/Well at a density of 50.000 cells. and treated for 96 h with 2ml of LC212-CM, LC212-CM pretreated with RNase and RPMI, as negative control. The cells were analyzed with morphological assay and Elisa assay.

### Elisa assay

TGF-β1amount in LC212-CM with and without RNase and in RPMI was detected by Human TGF-β1 Elisa Kit (Boster Immunoledder) in according to manufacturer’s instruction. All samples were processed in triplicate and averaged.

### Total RNA preparation

Total RNA was isolated from both cell culture supernatants and cells grown in adherence using a Total RNA purification plus kit and following manufacturer’s instructions (Norgen Biotek). Five mL of cell culture supernatants, from both LC212 and LC31 cells, were collected after 48 hours from cell seeding in a medium without FBS. Samples were immediately frozen at 80°C and then lyophilized. Total RNA of A549 cells was also isolated after treatment with TGF-β1[2ng/ml] and with human hsa-miR-21 antagomiR, used alone or in combination for 96h.

### TPCR array

RNA was reverse-transcribed using TaqMan MicroRNA Reverse Transcription kit (Applied Biosystems). cDNA was preamplified using TaqMan PreAmp Master Mix (Applied Biosystems). qRT-PCR was performed with an Applied Biosystem 7900HT Thermal Cycler using TaqMan human microRNA array (TaqMan Human microRNA Array A #4398977 and B v3.0 #442812; Applied Biosystems) according to manufacturer’s instructions. Data were then normalized calculating the ΔCt value for single miRNA against the average of specific controls for each card according to manufacturer's instructions. Differential expression analysis was performed according to ΔΔCt method and RQ ≥ 2 fold-change only were considered for further analysis. miRNAs clusters were generated through DIANA web tool mirPath v2.0 using miRBase MIMAT IDs (Release 21) remapped to the newest human genome assembly (GRCh38) to avoid duplicate entries present in the previous release. Only targets reported in TarBase database v7.0 were included in the clustering. False Discovery Rate (FDR) correction was applied to the original p-value and only clusters with corrected p-values < 0.05 were shown.

### miR-21 Real Time PCR analysis

Equal amounts of RNA was reverse-transcribed with a TaqMan Advanced miRNA cDNA Synthesis Kit (#A28007, Applied Biosystems) according to the manufacturer’s instructions. Real time PCR analysis of miR-21-3p and -5p was performed using a TaqMan Advanced miRNA Assay (#A25576,ID#477973_mir and #477975_mir, Applied Biosystems), using Applied Biosystems 7500 Fast thermal cycler. Each experiment was performed in triplicate. We normalized miR21 expression data using the levels of miR-16 (#A25576,ID#477860_mir, Applied Biosystems), because this miRNA is highly expressed and relatively invariant across samples. Data were then normalized calculating the ΔCt value for miR-21-3p and miR-21-5p against the miR-16. Differential expression analysis was performed according to ΔΔCt method and RQ ≥ 2 fold-change.

### Hsa-miR-21 antagomiR and Hsa-miR-21 Mimic transfection

Cells (200.000 cells) were seeded in flask 25 ml. Human hsa-miR-21 antagomiR was directly transfected into LC212 cells and A549 using Lipofectamine 2000 (#12566014, Invitrogen, USA) in according to the instructions provided by the manufacturer. After transfection, the LC212 cells were washed and the conditioned medium was collected and added to A549 cells for 48h.

The A549 cells (70.000 cells) were seeded in p6 well and was transfected with Hsa- miR21-3p-Mimic (#4464066, ID: MC12979) using Lipofectamine 2000 for 48h. The expression of EMT markers, Vimentin and E-cadherin, and SLUG and TWIST was performed with Real Time PCR assay.

### Western blot for vimentin, SMAD2/3, E-cadherin, EpCAM

Western blot was performed in according to standard procedures. Rabbit monoclonal antibody EpCAM (clone E6V8Y, 1:1000; Cell Signaling Technology, Danvers, MA, USA), mouse monoclonal antibody Vimentin (clone V9, 1:1000, Abcam, Cambridge, UK), rabbit monoclonal antibody against GAPDH (clone 6C5, 1:1000, Santa Cruz Biotechnology, Heidelberg, Germany), mouse monoclonal antibody E-Cadherin (clone 5H9, 1:500, Abcam, Cambridge, UK) were used. Detection was performed by HRP-conjugated anti-mouse (Cell Signaling Technology, 1:2000) or HRP-conjugated anti-rabbit (Cell Signaling Technology, 1:2000) antibodies. Immune complexes were visualized by an enhanced chemiluminescence system (Western Bright ECL, Advansta Corporation, Menlo Park, CA, USA). GAPDH was used as loading control. The image analysis was performed by ImageJ software (http://rsbweb.nih.gov/ij/). Results represent the means (±SEM) of three independent experiments performed in triplicate. P-values were determined by using t-tests and p<0.05 was considered to be statistically significant.

### Statistical analysis

Values are shown as the mean ± S.E.M. of measurements of at least three independently performed experiments to avoid possible variation of cell cultures. Student’s t test was employed, and p<0.05 was considered to be statistically significant.

## Results

### Establishment and phenotypic analysis of the NSCLC LC212 primary cell line

**Fig 1:**
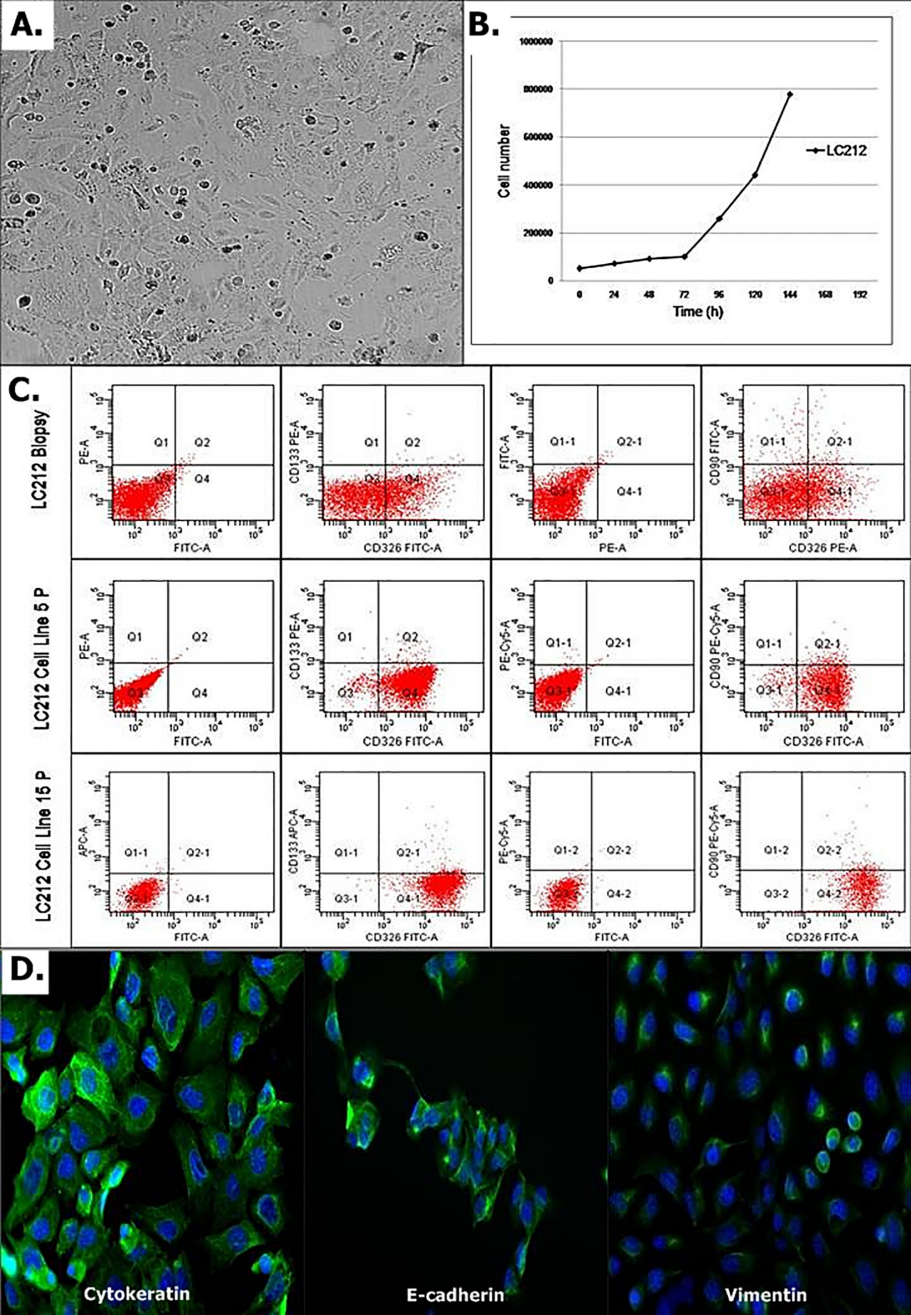
Characterization of LC212 primary cell line. (A) Morphology of LC212 cells cultured in RPMI1640 plus 10% FBS showing the epithelioid shape of cells; (B) growth curve of LC212 cell line with a doubling time of 86 hours. Scale bar = 50μm. (C) Markers expression by flow cytometry in LC212 biopsy, LC212 5P and LC212 15P;(C) cytokeratin, e-cadherin and vimentin immunofluorescence assay in LC212 15P. Scale bar = 50μm.

After enzymatic digestion of tumor, the LC212 cell suspension was cultured in three different combinations of media as described in the materials and methods. After 4–5 days of culture, lung tumor cells adhered on the plate surface only in RPMI medium with 10% FBS. The adherent cultured cells showed polygonal morphology that was maintained through repeated passages of culture. This primary cell line was considered as a stabilized cell line after 15–passages of culture. The growth curve analysis showed that LC212 cell line had a doubling time of 48 hours. During growth, LC212 cells secreted tridimensional structures resembling granules in suspension. (Fig 1A and 1B).

LC212 cells at the day of surgical collection and after five (5P) and fifteen passages (15P) of culture were analyzed by flow cytometry to evaluated the expression of CD326 (EpCam), CD90, and CD133. The results showed that cells positive for CD326 were 25.2%, 85.2%, 92.3% of the total cell population in the surgical biopsy, 5P and 15P, respectively. These data suggest a progressive enrichment of the initial culture for epithelial cells. CD90 and CD133 expression were 2.5% and 0.3% in biopsy, 1.9% and 0.6% at 5P of culture and 1.3% and 0.9% at 15P of culture (Fig 1C). All cells were negative for the CD45 leukocyte marker.

To further characterize the phenotype of the LC212 cell line, we performed also an immunofluorescence analysis for cytokeratin, e-cadherin and vimentin. cytokeratin and e-cadherin were highly expressed and uniformly distributed in all cells. On the contrary, vimentin was weakly expressed and it was contained in perinuclear vesicles (Fig 1D).

### LC212-CM induced changes in A549 cell morphology and phenotype

**Fig 2:**
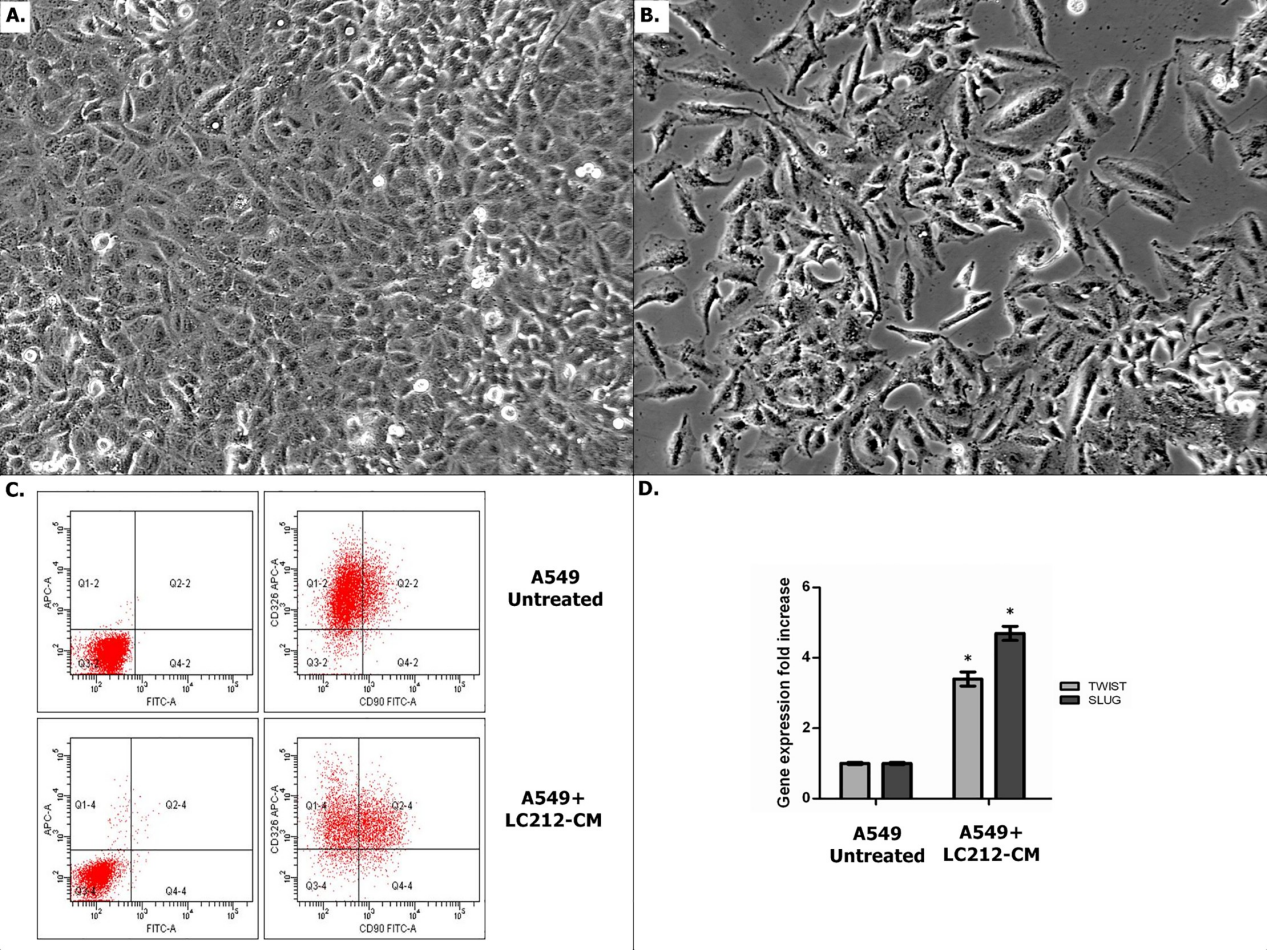
Effect of LC212-CM on A549 cell line. (A,B) Optical microscopy of A549 cells showed the morphological effect induced by LC212-CM. LC212-CM induced morphological changes resembling mesenchymal cells-like morphology; conversely, no shape changes have been observed if standard medium were used. Scale bar = 50μm. (C) EMT characterization by flow cytometry evidenced the over-expression of CD90 and down-regulation of CD326 on A549 treated with LC212-CM respect to untreated A549 cells; (D) TWIST and SLUG Real Time expression in A549 cells treated with LC212-CM. The data showed an up-regualtion of these genes respect to untreated A549 cells. * p<0.03 compared to the untreated cells.

We next evaluated the effects of the LC212-CM on the A549 lung cancer cell line, which we have previously shown to undergo EMT following treatment with TGF-β1 [9]. We found that LC212-CM led to morphological changes in A549 cells. In particular, untreated A459 cells had a clear epithelial morphology (Fig 2A), while A549 cells treated with LC212-CM showed an elongated shape resembling fibroblast like profiles (Fig 2B).

These morphological changes were also associated with a shift from epithelial to mesenchymal marker expression. In fact, cytometric analyses showed that A549 cells, seeded in standard medium, displayed an average expression of CD326 and CD90 markers of approximately 80% and 11.60%, respectively. When A549 were treated with LC212-CM, we observed an increase of CD90 expression with a mean percentage of 32.60%, and a decrease of CD326 expression with a mean percentage of 68.50% (Fig 2C). Since EMT is also characterized by an increased expression of transcriptional factors such as SLUG and TWIST [23], we analysed the levels of these genes by real time PCR. The results showed increased SLUG and TWIST levels of expression in A549 cells treated with LC212-CM as compared to untreated cells. (Fig 2D)

### miRNA profiling in LC212 cell line

We investigated the possible role of TGF-β1 and miRNA in the ability of the LC212-CM to induce EMT.

**Fig 3:**
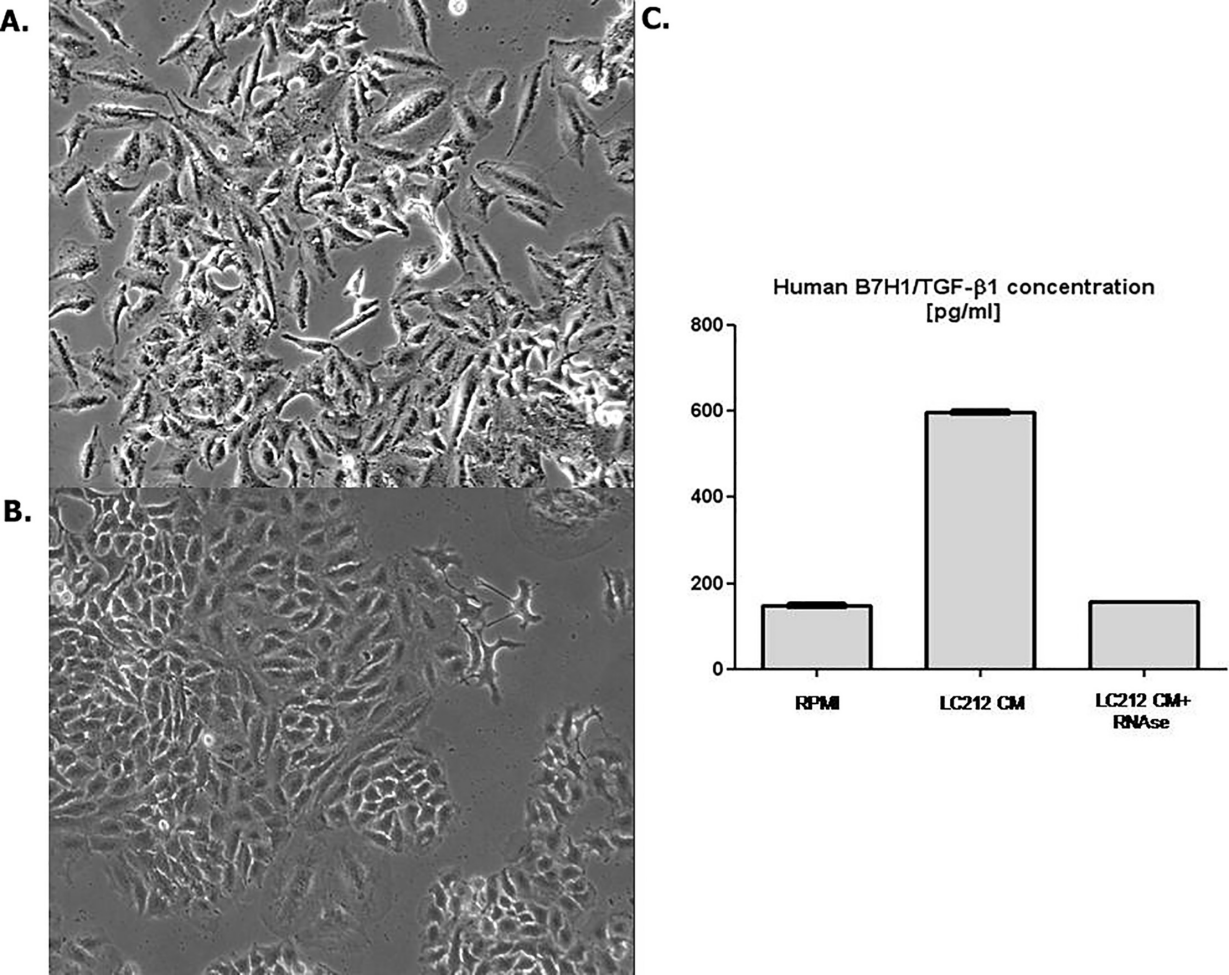
Characterization of LC212-CM. (A) A549 cell line shows a mesenchymal morphology with LC212 CM. (B) A549 cells conserved the epithelial morphology after treatment of conditioned medium with RNAse. (C) Human B7H1/TGF-β1 ELISA Assay. The concentration of activated human TGF-β1 was higher in the LC212-CM respect the same medium treated with RNAse.

LC212-CM was collected in presence or in absence of RNAse. A549 cells treated with LC-212 CM collected in presence of RNAse conserved the epithelial morphology, differently to the EMT phenotype of cells exposed to LC212-CM without RNAse (Fig 3A and 3B). The TGF-β1 amount in LC212-CM was measured by ELISA. We found that the levels of TGF-β1 were significantly higher in LC212-CM than those found in standard control medium. Surprisingly, RNAse treatment of LC212 cells resulted in a significant decrease in the levels of TGF-β1 thus suggesting a role of miRNA in the regulation of this growth factor in LC212 cells (Fig 3C).

**Fig 4:**
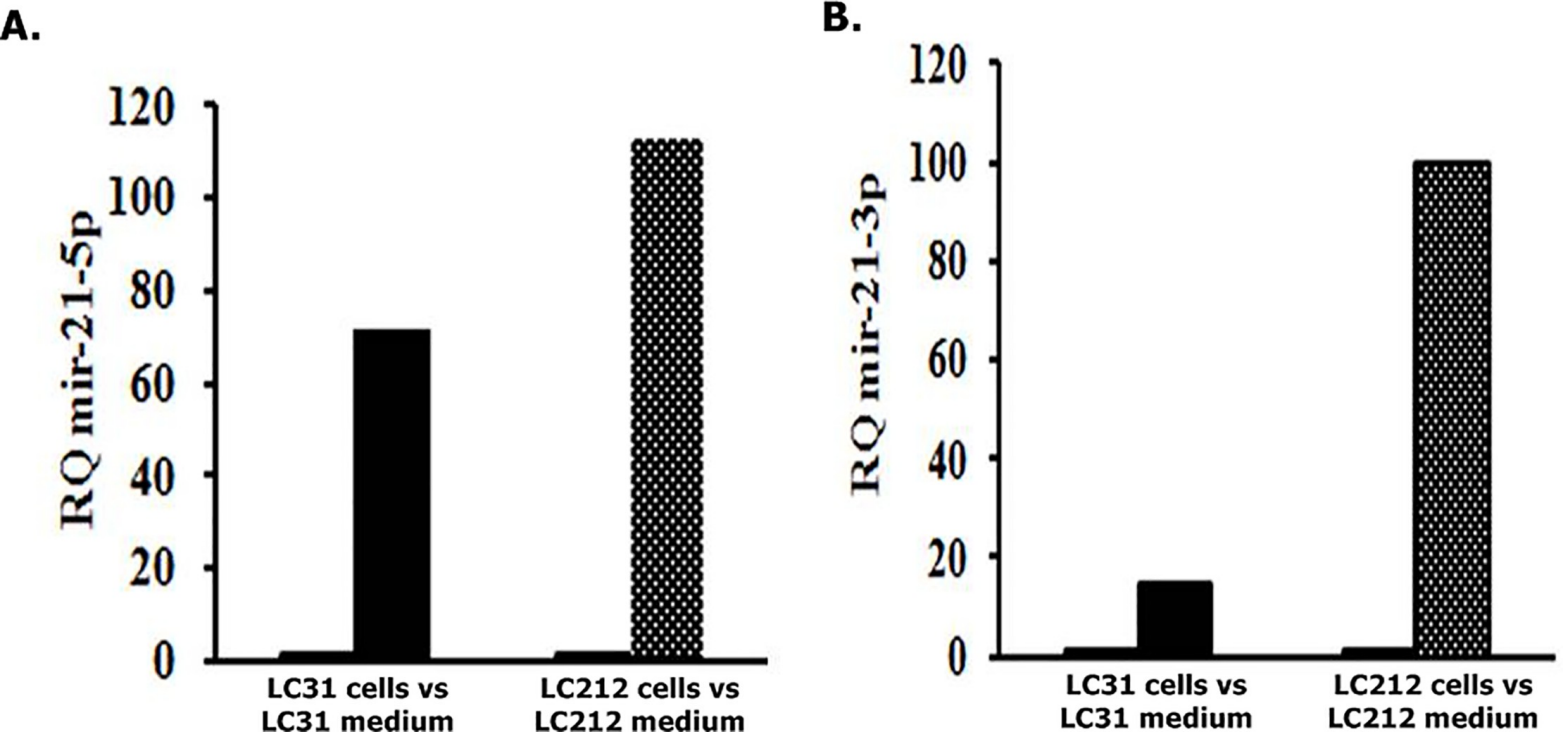
Relative expression of hsa-mir-21-5p and hsa-mir-21-3p. **(A)** Relative quantification of miR-21-5p in LC212 cells vs LC212CM and LC31 cell vs LC31 CM; **(B)** Relative quantification of hsa-mir-21-3p in LC212 cells vs LC212CM and LC31 cell vs LC31 CM. hsa-mir-21-3p was significantly most abundantly released outside the LC212 than the LC31 cells.

Overall, these findings suggest that EMT induction could be due to specific miRNAs in cooperation with TGF-β1. To verify this hypothesis, both cellular and extracellular miRNA expression profiling were performed in LC212 cell line using TaqMan Human MicroRNA Arrays. As control, we used LC31 cells which were found not to induce EMT in A549 cells (S4 Fig.) [22]. A total of 768 miRNAs, present in the array, were analyzed in each cell model. A total of 409 and 413 miRNAs were found expressed in LC212 and LC31 cells, respectively. We identified 261 miRNAs expressed in both cell lines. A set of 82/261 were found differentially expressed at least with 2-fold change; whereas 179 miRNAs were filtered out by the threshold applied. Considering the miRNAs differentially expressed, a functional analysis was performed using the DIANA-mirPath 2.0 tool and in particular the software TarBase, which uniquely clusters those miRNAs whose targets are experimentally validated [24]. The obtained clusters were filtered based on their significance (FDR corrected p < 0.05). Target genes resulted grouped into functional categories (KEGG_pathways and GOterm, S1 Table and S2 Table) associated with cancer phenotype in LC212 and LC31 cell lines, considering both cellular and extracellular miRNAs. However, from the analysis of the main KEGG pathways appeared that miR-21 was differently present as both cellular and extracellular component in LC212 respect with LC31 cells. Comparing the abundance of the two isoforms of hsa-miR-21 (3p and 5p) released by the two cell lines in the culture supernatants it was found that hsa-miR-21-3p was significantly most abundantly released outside LC212 than LC31 cells (Fig 4A and 4B).

### hsa-miR-21 silencing

The expression and modulation of hsa-miR-21 was evaluated by using antagomiR strategy.

**Fig 5:**
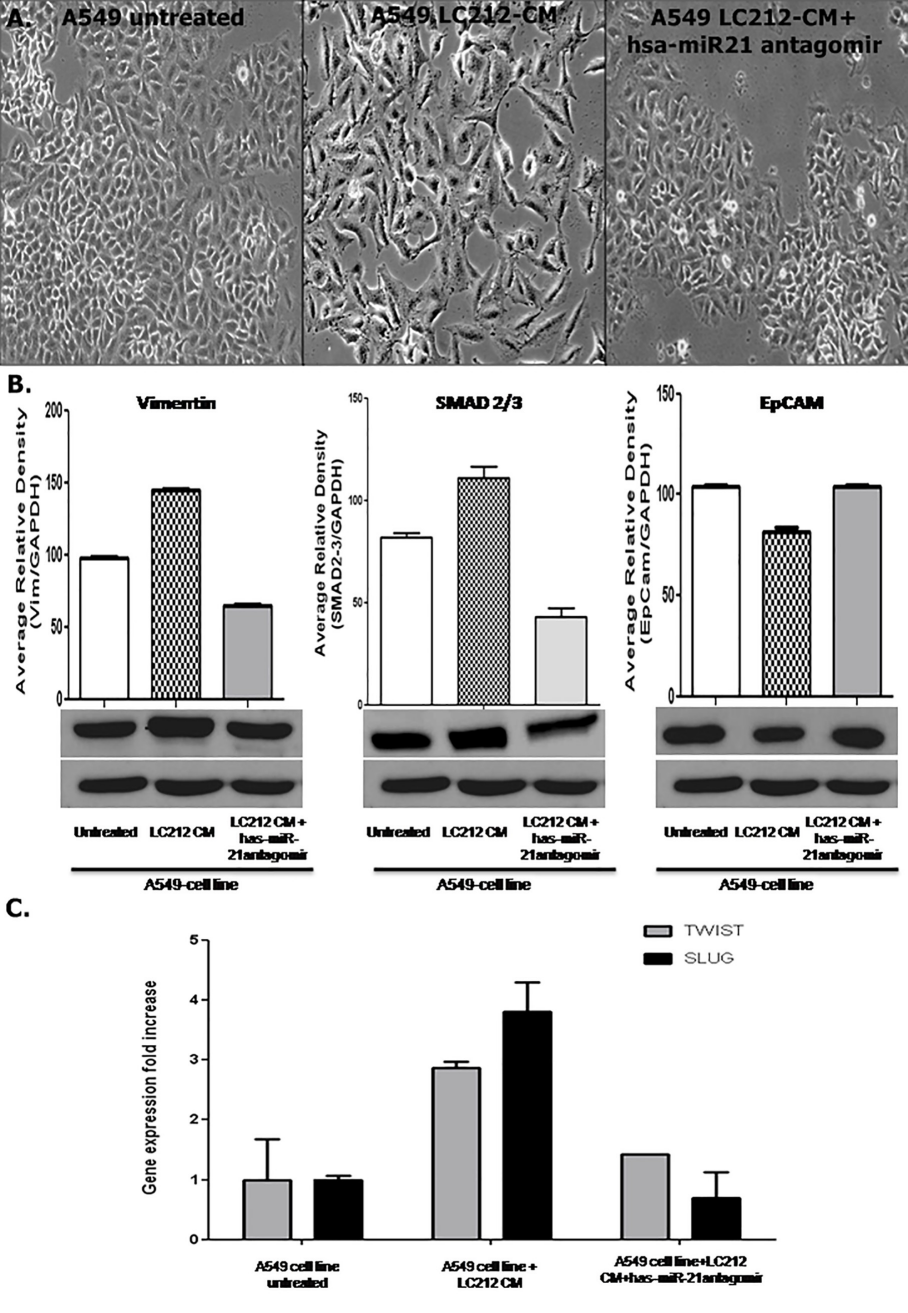
Hsa-miR-21 antagomir effect on EMT phenotype. (A) No morphological change was detectable in A549 cell line untreated and A549 cell line treated with LC212-CM plus hsa-miR-21 antagomir respect to A549 cells with LC212-CM. Scale bar = 50μm. (B) EMT characterization by Western Blot analyses evidenced the over-expression of Vimentin and SMAD 2/3 and the down-regulation of E-Cadherin in A549 treated with LC212-CM respect to untreated A549 cells and A549 treated with LC212-CM plus hsa-miR-21 antagomir. S2 Fig. Blot images presented in the manuscript uncropped and unadjusted in the Fig 5. (C) TWIST and SLUG Real Time analysis. A549 cells treated with LC212-CM showed an up-regulation of these genes respect to untreated A549 cells and A549 treated with LC212-CM plus hsa-miR-21 antagomir.

Transfection of LC212 cells with hsa-miR-21 antagomiR was performed and the conditioned medium was analyzed by real-time-PCR. The data evidenced a very significant downregulation of the hsa-miR-21 following treatment with hsa-miR-21 antagomiR (S1 Fig). Treatment of A 549 cells with LC212-CM plus hsa-mir-21 antagomiR did not induce morphological changes associated with the EMT (Fig 5A). To further investigate if hsa-miR-21 was involved in EMT induction, a western blot analysis of vimentin, SMAD2/3 and EpCam was performed. The analysis showed an up-regulation of vimentin and SMAD 2/3, mesenchymal markers, in A549 cells treated with LC212-CM and a down regulation of the same proteins when hsa-miR-21 antagomiR was added to the culture medium. Differently, the expression of EpCam was higher in A549 untreated and treated with LC212-CM plus hsa-miR-21 antagomiR than in those treated with LC212-CM (Fig 5B). Moreover, LC212-CM plus hsa-miR-21 antagomiR did not induce changes in the levels of SLUG and TWIST mRNAs in A549 cells as compared to untreated cells, whereas an over expression of both genes was observed in cells treated with LC212-CM (Fig 5C). Finally, deregulation of EMT markers was observed after transfection assay with Hsa-mir-21 Mimic on A549 cells. The results were showed in supplementary data (S5 Fig.) These data confirmed our hypothesis that hsa-miR-21 is involved in EMT.

### Hsa-miR-21 and TGFβ1 induced EMT in A549 lung cancer cell line

**Fig 6:**
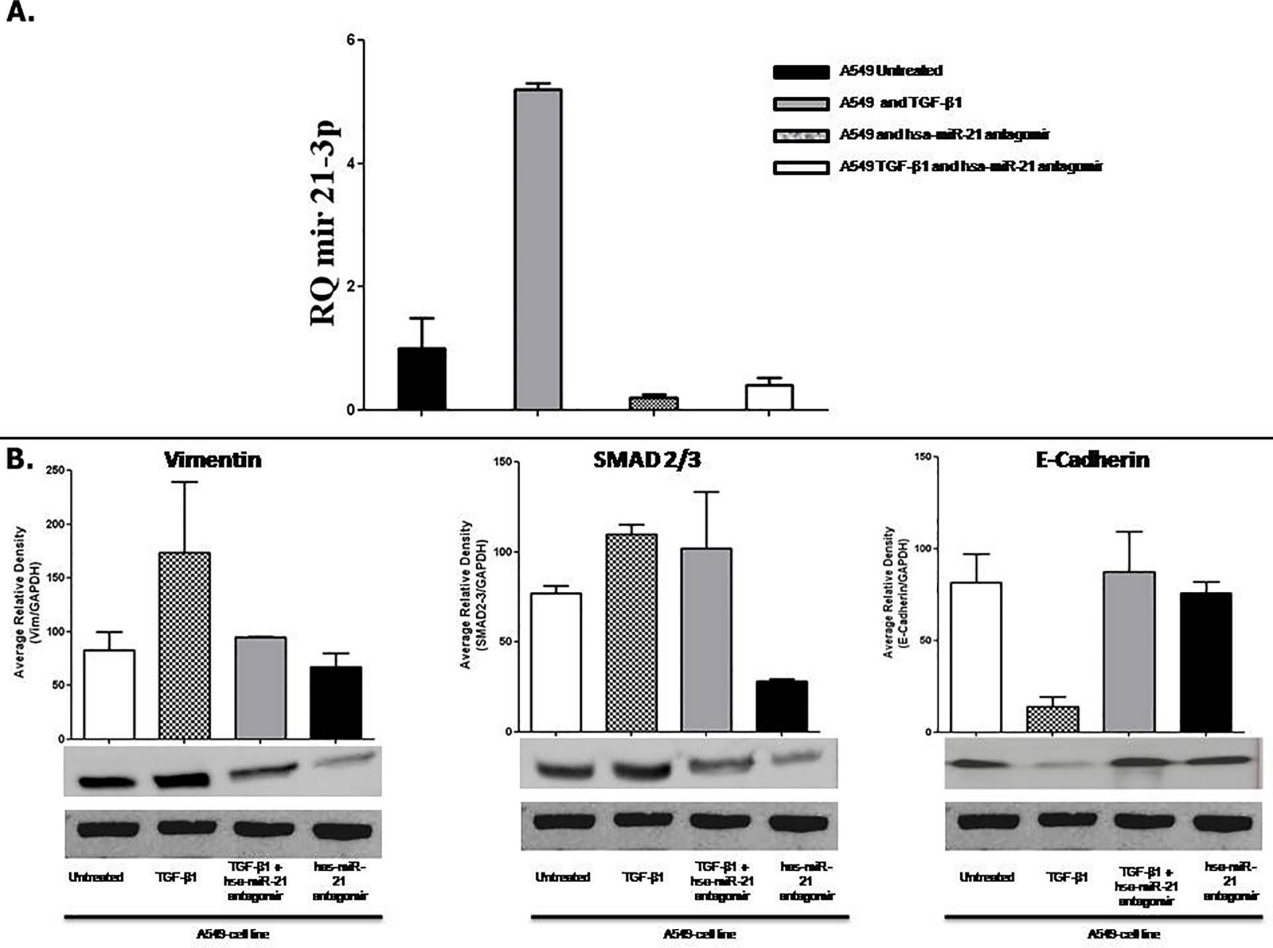
(A) **Relative expression of hsa-mir-21-3p.** The data showed a down-regulation of has-miRna in A549 cells treated with TGF-**β**1 plus hsa-miR-21 antagomir and hsa-miR-21 antagomir alone, compareted to A549 cells with TGF-**β**1 alone. (B) **EMT characterization of A549 treated with TGF-β1 by Western Blot analyses.** The data showed the over-expression of Vimentin and SMAD 2/3 and the down regulation of E-Cadhern in A549 cell line treated with TGF-**β**1. The results showed a reversion of Vimentin and SMAD 2/3 to basal level after treatment with hsa-miR-21 antagomir. S3 Fig. Blot images presented in the manuscript uncropped and unadjusted in the Fig 6.

Finally, we addressed whether endogenous hsa-miR-21 might be involved in the EMT of A549 cells. In fact, an in silico analysis using the miRmine-Human miRNA Expression Database suggested the expression of hsa-miR-21 in A549 cells. Therefore, we examined the expression of hsa-miR-21 in A549 cells after treatment with hsa-miR-21 antagomiR and TGF-**β**1. The levels of hsa-miR-21 were significantly increased in A549 cells following treatment with TGF-**β**1. Treatment of hsa-miR-21 antagomiR in the presence or absence of TGF-**β**1 decreased of more than 90% the levels of hsa-miR-21 as compared to A549 cells treated with TGF-**β**1 (Fig 6A).

Hsa-miR-21 antagomiR was able to in part counteract the effects of TGF-**β**1 on the expression of mesenchymal and epithelial markers in A549 cells. In particular, it was able to prevent the induction of vimentin and the decrease in e-cadherin, whereas the effects on SMAD 2/3 were less evident (Fig 6B).

## Discussion

EMT is a biological process through which epithelial cells acquire high motility and invasion. Several studies showed that the EMT mediated by TGF**β**-1 plays a key role in tumor progression, drug resistance and metastasis [6,9,13,25]. In cancer, TGF**β**-1 has been shown to have double role. In the early stages of tumorigenesis, TGF**β**-1 acts as a tumor suppressor, whereas in the later stages this factor assumes a function of tumor promoter [26,27]. In this respect, we have previously demonstrated the role of TGF-**β**1 in the regulation of EMT in lung cancer cells. In primary lung cancer cells, the induction of EMT by TGF**β**-1 exposure was associated with a reduction in cell-cell adhesion and expression of mesenchymal markers, such as Slug, Twist and **β**-catenin, as well as an upregulation of the expression of stem cell markers[9,12]. Moreover, same studies highlighted the association between EMT and acquired drug- resistance to EGFR tyrosine kinase inhibitors. In this respect, novel treatment strategies are being developed to overcome or prevent the acquisition of mesenchymal pattern. [28]

Other factors that play a key role in EMT process are miRNA. Previous reports demonstrated that miRNAs can regulate EMT in cancer development [29,30]). In particular, numerous miRNAs modulate expression of genes involved in cell adhesion and tumor microenvironment to promote dissociation of cancer cells from the primary tumor. MicroRNAs can either repress or stimulate migration and invasion of cancer cells to modify integrity of epithelial architecture. For example, in NSCLC overexpression of miR-574-5c induces loss of E-cadherin-mediated cell-adhesion by downregulation of **β**-catenin. [31,32]

In this study, we hypothesize a cooperation between TGF**β**-1 and miRNAs and, in particular, hsa-miR-21 in the regulation of EMT in lung cancer cells.

Several studies demonstrated that the expression of different proteins involved in the TGF**β** signaling pathways, including members of the TGF**β**-1 family of growth factors and receptors, may be influenced by miRNAs. Indeed, in gastric cancer cells, hsa-miR-106b and hsa-miR-25 were found to be upregulated and correlated with the loss of tumor suppressor activity of TGF**β**-1 signaling [33]. In addition to tumorigenesis, TGF**β**-1 signaling and miRNAs have been shown to cooperate in the development of several organs, for example in regulation of fibrogenesis, in the liver and in the lung hsa-miR-21 targeting the negative regulator SMAD7 can also enhance TGF**β** signaling [34,35].

Our hypothesis of a cooperation of TGF**β**-1 and miRNA in inducing EMT in lung cancer cells is supported by different observations. First, we found that LC212-CM led to morphological changes in A549 cells supported both from cytometric data and from overexpression of SLUG and TWIST, as EMT markers. Moreover, we found that the levels of TGF**β**-1 in the conditioned medium of LC212 cells were significantly lower when the medium was collected in the presence of RNAse. In addition, treatment of LC212 cells and A549 cells with hsa-miR-21 antagomiR significantly reduced the levels of mesenchymal markers. On the contrary, treatment of A549 cells with hsa-miR-21 Mimic showed overexpression of Vimentin, SLUG and TWIST as EMT markes. Given that treatment with TGF**β**-1 was able to induce hsa-miR-21 in A549 cells, we might hypothesize a complex interplay between these two factors that are able to induce the expression of each other thus reinforcing autocrine and paracrine loops that sustain the EMT process.

Evidence suggest that hsa-miR-21 has a relevant role in the pathogenesis and progression of different tumor types, including lung cancer([19–21]. Lin and colleagues demonstrated that hsa-miR-21 as regulator of SMAD7, may be used as a predictor for the prognosis of the NSCLC after carboplatin treatment [36]). Other studies have reported that in HCC the aberrant expression the hsa-miR-21 can contribute to growth and spread by modulating PTEN expression and PTEN dependent pathways involved in cell growth, migration and invasion [37]. Finally, hsa-miR-21 has been described to regulate the biological behavior of cholangiocarcinoma and breast cancer by inducing EMT [38–40].

In conclusion, we demonstrated a cooperative activity of TGF-**β**1 and hsa-miR21 in regulating the EMT processes in lung cancer cells. Our findings confirm that hsa-miR-21 could be considered as a potential oncogene in NSCLC able to induce tumor progression, This hipothesys is supported also by previous studies that put in light the functional role of hsa miR-21 in NSCLC cell line during apoptosis and acquired drug resistence [41,42] Therefore, both TGF**β**-1 and hsa-miR-21 might represent relevant targets for therapeutic intervention in NSCLC.

## Supporting information

S1 Table Mirna and gene profiling with software TarBase. miRNAs and their target genes expressed by the cells or released in the supernatants of LC212 and LC31 cell lines.
(DOCX)

S2 Table Gene Ontology of miRNA target genes. miRNA target genes expressed by the cells or released in the supernatants of LC212 and LC31 cell lines.
(DOCX)

S1 Fig Relative amount of hsa-mir-21-3p in LC212 CM. Relative quantification of hsa miR-21-3p relased in LC212-CM and in LC212-CM treated with hsa-miR-21 antagomir.
(TIF)

S2 Fig Blot images presented in the manuscript uncropped and unadjusted in the Fig 5. (TIF)

S3 Fig Blot images presented in the manuscript uncropped and unadjusted in the Fig 6. (TIF)

S4 Fig Panel relative to experiment control with LC31-CM. **(A)**A549 cells untreated (B) A549 cells conserved the epithelial morphology after treatment of conditioned medium derived to LC31 cell line for 48h. Morfological and Immunofluorescence assay.
(TIF)

S5 Fig Relative expression of EMT markers in A549 cells after Mimic transfection. Relative quantification of EMT markes showed the over-expression of Vimentin, SLUG and TWIST and downregulation of E-Cadherin after transfection with hsa-mir-21-3p Mimic (used 60 pMol and 90 pMol for 48h) on A549 cells.
(TIF)
