# Supplementary material for: Conditioned medium of primary lung cancer cells induces EMT in A549 lung cancer cell line by TGF-ß1 and miRNA21 cooperation
Source: PLoS One. 2019 Jul 25;14(7):e0219597. doi: 10.1371/journal.pone.0219597 (PMC6657837; doi:10.1371/journal.pone.0219597)
Supplement: S1 Table — miRNAs and their target genes expressed by the cells or released in the supernatants of LC212 and LC31 cell lines. (DOCX) [file pone.0219597.s001.docx]

**Supplementary material 1. miRNAs, and their relative target genes, specifically expressed by the cells or released in the supernatants of LC212 and LC31 cell lines.**

| **Term (KEGG_Pathway)** | **Count** | **miRNA\|TarBase** | **Count** | **PValue** | **Genes** | | | | | |
| --- | --- | --- | --- | --- | --- | --- | --- | --- | --- | --- |
| **Cell cycle (hsa04110)** | 5 | miR-21  miR-572  miR-376a-3p  miR-34b-3p  miR-503 | 18 | 5.144918E-11 | E2F1 | SMC1A | CDK4 | E2F2 | CDC14A | STAG2 |
|  |  |  |  |  | WEE1 | CDK6 | CHEK1 | CCND1 | CCNE2 | SKP2 |
|  |  |  |  |  | MYC | TTK | TGFB2 | CCNE1 | CDKN1A | CDC25A |
| **Pathways in cancer (hsa05200)** | 7 | mirR-21  miR-572  miR-147a  miR-503  miR-34b-3p  miR-223-3p  miR-200c-3p | 42 | 1.593502E-10 | STAT3 | BID | CHUK | PTK2 | MMP9 | CCNE1 |
|  |  |  |  |  | E2F1 | TCF4 | BCL2 | CCND1 | MSH2 | FN1 |
|  |  |  |  |  | TGFBR1 | APC | PLD1 | MSH6 | PIK3R1 | CDKN1A |
|  |  |  |  |  | ERBB2 | WNT1 | EGFR | CCNE2 | FAS | Il6 |
|  |  |  |  |  | CDK4 | WNT5A | APPL1 | SKP2 | TGFB2 | VEGFA |
|  |  |  |  |  | E2F2 | TCF7L1 | CDK6 | MYC | EP300 | PTEN |
|  |  |  |  |  | TGFBR2 |  |  |  |  |  |
| **p53 signaling pathway (hsa04115)** | 5 | miR-21  miR-503  miR-572  miR-1285-3p  miR-34b-3p | 17 | 3.700236E-05 | CCNG1 | CDK4 | BID | THBS1 | CDK6 | CHEK1 |
|  |  |  |  |  | TP53 | APAF1 | CCND1 | CCNE2 | SESN1 | MDM4 |
|  |  |  |  |  | FAS | SERPINB5 | CCNE1 | CDKN1A | PTEN |  |
| **PI3K-Akt signaling pathway (hsa04151)** | 5 | miR-21  miR-147a  miR-151a-3p  miR-34b-3p  miR-503 | 23 | 4.851066E-05 | TSC1 | ITGB8 | CDK4 | THBS1 | MCL1 | BCL2 |
|  |  |  |  |  | EGFR | TLR4 | CDK6 | PTK2 | BRCA1 | CCND1 |
|  |  |  |  |  | VEGFA | PTEN | SGK3 | COL5A2 | GNB4 | CDKN1A |
|  |  |  |  |  | CCNE2 | MYC | PIK3R1 | CCNE1 | PDGFD | CDKN1A |
| **Adherens junction (hsa04520)** | 3 | miR-200c-3p  miR-661  miR-224-5p | 5 | 0.01943897 | TCF7L1 | VCL | CDC42 | EP300 | PVRL1 |  |
|  |  |  |  |  |  |  |  |  |  |  |
| **HIF-1 signaling pathway (hsa04066)** | 4 | miR-21  miR-572  miR-34b-3p  miR-147a | 11 | 0.02622181 | STAT3 | ERBB2 | BCL2 | EGFR | TLR4 | PDHA2 |
|  |  |  |  |  | PIK3R1 | CDKN1A | PFKFB2 | MKNK2 | VEGFA |  |

**Increased in LC212 cells**

**Increased in LC212 supernatant**

| **Term (KEGG_Pathway)** | **Count** | **miRNA\|TarBase** | **Count** | **PValue** | **Genes** | | | | | |
| --- | --- | --- | --- | --- | --- | --- | --- | --- | --- | --- |
| **Pathways in cancer (hsa05200)** | 8 | miR-200c-3p  miR-107  miR-98  miR-34b-3p  let-7f-5p  miR-182-5p  miR-330-3p  miR-21 | 50 | <1E-16 | STAT3 | APC | EGFR | MYC | CCNE1 | BID |
|  |  |  |  |  | E2F1 | WNT1 | APPL1 | NFKB2 | FN1 | TCF4 |
|  |  |  |  |  | TGFBR1 | WNT10B | CDK6 | MMP9 | CDKN1A | PLD1 |
|  |  |  |  |  | ERBB2 | WNT5A | MITF | MSH2 | BIRC3 | CDKN2B |
|  |  |  |  |  | CDK4 | TCF7L1 | PTK2 | EGLN3 | VEGFA | SKP2 |
|  |  |  |  |  | E2F2 | ARNT | FZD10 | PIK3R1 | PTEN | HIF1A |
|  |  |  |  |  | NRAS | BCL2 | CCND1 | FAS | FOXO1 | TGFB2 |
|  |  |  |  |  | CRKL | CDKN1B | MSH6 | LAMC1 | IL6 | EP300 |
|  |  |  |  |  | TRAF1 | TGFBR2 |  |  |  |  |
| **Cell cycle (hsa04110)** | 8 | miR-376a-3p  miR-107  hsa-miR-98  miR-34b-3p  let-7f-5p  miR-885-5p  miR-330-3p  miR-21 | 22 | 8.33589E-11 | CDK7 | E2F2 | CDKN1B | CDK6 | TTK | CDKN1A |
|  |  |  |  |  | E2F1 | CCNA2 | MCM5 | CCND1 | CDC23 | CDC25A |
|  |  |  |  |  | SMC1A | CDK2 | STAG2 | SKP2 | TGFB2 | CDK4 |
|  |  |  |  |  | DBF4 | CDKN2B | MYC | CCNE1 |  |  |
| **Transcriptional misregulation**  **in cancer(hsa05202)** | 6 | miR-652-3p  miR-98  let-7f-5p  miR-182-5p  miR-1285-3p  miR-126-5p | 15 | 1.03058E-07 | HMGA2 | HOXA9 | CDKN1B | SLC45A3 | TP53 | MYC |
|  |  |  |  |  | KLF3 | BMP2K | REL | CDKN1A | BIRC3 | FOXO1 |
|  |  |  |  |  | IL6 | TRAF1 | MEIS1 |  |  |  |
| **HIF-1 signaling pathway (hsa04066)** | 6 | miR-107  miR-98  miR-34b-3p  miR-182-5p  miR-330-3p  miR-21 | 24 | 0.0002372919 | STAT3 | BCL2 | IFNGR1 | EGLN3 | CDKN1A | IL6 |
|  |  |  |  |  | ERBB2 | CDKN1B | RPS6KB2 | PIK3R1 | PFKFB2 | PDHB |
|  |  |  |  |  | LTBR | EGFR | HIF1A | EP300 | MKNK2 | RPS6KB1 |
|  |  |  |  |  | ARNT | TLR4 | PDHA2 | EIF4E2 | VEGFA | IL6R |
| **PI3K-Akt signaling pathway (hsa04151)** | 4 | miR-107  miR-34b-3p  let-7f-5p  miR-21 | 22 | 0.0005923302 | TSC1 | THBS1 | CDK6 | MYC | CDKN1A | COL5A2 |
|  |  |  |  |  | MYB | BCL2 | PTK2 | PIK3R1 | VEGFA | GNB4 |
|  |  |  |  |  | ITGB8 | EGFR | BRCA1 | CCNE1 | PTEN | SGK3 |
|  |  |  |  |  | CDK4 | TLR4 | CCND1 | PDGFD |  |  |
| **p53 signaling pathway (hsa04115)** | 6 | miR-107  miR-34b-3p  let-7f-5p  miR-885-5p  miR-1285-3p  miR-21 | 16 | 0.001450659 | CCNG1 | CDK4 | BID | THBS1 | CDK2 | CDK6 |
|  |  |  |  |  | TP53 | APAF1 | CCND1 | SESN1 | MDM4 | FAS |
|  |  |  |  |  | SERPINB5 | CCNE1 | CDKN1A | PTEN |  |  |

**Increased in LC31 cells**

| **Term (KEGG_Pathway)** | **Count** | **miRNA\|TarBase** | **Count** | **PValue** | **Genes** | | | | | |
| --- | --- | --- | --- | --- | --- | --- | --- | --- | --- | --- |
| **Pathways in cancer (hsa05200)** | **5** | miR-155-5p  miR-363-3p  miR-504  miR-372  miR-449b-5p | 38 | 5.699169E-06 | GSK3B | STAT3 | NFKB1 | SPI1 | CDK4 | SMAD2 |
|  |  |  |  |  | CDK2 | BAX | MDM2 | ETS1 | SMAD3 | EGFR |
|  |  |  |  |  | CDKN2A | KRAS | CDK6 | Hif1a | MLH1 | SMAD4 |
|  |  |  |  |  | MSH6 | CTNNA1 | COL4A2 | MSH2 | RAC1 | FGF2 |
|  |  |  |  |  | NKX3-1 | CDKN1A | VEGFA | CSF1R | FGF7 | JUP |
|  |  |  |  |  | Spi1 | APC | WNT5A | E2F2 | RHOA | CTNNB1 |
|  |  |  |  |  | FAS |  |  |  |  |  |
| **PI3K-Akt signaling pathway (hsa04151)** | **7** | miR-155-5p  miR-615-3p  miR-377-3p  miR-372  miR-449b-5p  miR-23a-3p  miR-876-3p | 32 | 8.402651E-06 | GSK3B | NFKB1 | CDK4 | THBS1 | FGF7 | GNB4 |
|  |  |  |  |  | CDC37 | MCL1 | CDK2 | PCK2 | FGF2 | EIF4E2 |
|  |  |  |  |  | KRAS | CDK6 | RHEB | ITGB5 | FOXO3 | IL6R |
|  |  |  |  |  | FLT1 | PDK1 | YWHAZ | RAC1 | PAK1 | COL4A2 |
|  |  |  |  |  | PKN2 | CDKN1A | VEGFA | CSF1R | EGFR | PPP2R2A |
|  |  |  |  |  | Csf1r | ITGB4 |  |  |  |  |
| **p53 signaling pathway (hsa04115)** | **5** | miR-483-3p  miR-363-3p  miR-504  miR-372  miR-449b-5p | 7 | 5.741087E-05 | CDK4 | BAX | CDK6 | FAS | CDKN1A | BBC3 |
|  |  |  |  |  | MDM2 |  |  |  |  |  |
|  |  |  |  |  |  |  |  |  |  |  |
| **Cell cycle (hsa04110)** | **5** | miR-155-5p  miR-483-3p  miR-363-3p  miR-372  miR-449b-5p | 16 | 0.003356094 | SMAD2 | E2F2 | CDK2 | SMAD3 | STAG2 | WEE1 |
|  |  |  |  |  | CDK6 | SMAD4 | YWHAZ | CDKN1A | PRKDC | PLK1 |
|  |  |  |  |  | GSK3B | CDK4 | CDKN2A | CDC25A |  |  |
| **TGF-beta signaling pathway (hsa04350)** | **2** | miR-155-5p  miR-483-3p | 7 | 0.01522968 | SMAD2 | THBS1 | SMAD3 | RHOA | SMAD4 | SMAD5 |
|  |  |  |  |  | SMAD1 |  |  |  |  |  |

**Increased in LC31 supernatant**

| **Term (KEGG_Pathway)** | **Count** | **miRNA\|TarBase** | **Count** | **PValue** | **Genes** | | | | | |
| --- | --- | --- | --- | --- | --- | --- | --- | --- | --- | --- |
| **Adherens junction (hsa04520)** | 3 | miR-155-5p  miR-483-3p  miR-519-3p | 13 | 4.695017E-07 | CTNND1 | SMAD2 | SMAD3 | EGFR | RHOA | TJP1 |
|  |  |  |  |  | SMAD4 | CTNNB1 | CTNNA1 | PTPRJ | RAC1 | SSX2IP |
|  |  |  |  |  | YES1 |  |  |  |  |  |
| **TGF-beta signaling pathway (hsa04350)** | 2 | miR-155-5p  miR-483-3p | 7 | 3.176525E-06 | SMAD2 | THBS1 | SMAD3 | RHOA | SMAD4 | SMAD5 |
|  |  |  |  |  | SMAD1 |  |  |  |  |  |
| **Pathways in cancer (hsa 05200)** | 2 | miR-155-5p  miR-223-3p | 35 | 0.000798038 | SPI1 | CDK4 | SMAD2 | E2F2 | Spi1 | APC |
|  |  |  |  |  | SMAD4 | CTNNB1 | MSH6 | CTNNA1 | COL4A2 | MSH2 |
|  |  |  |  |  | CDK2 | Csf1r | ETS1 | SMAD3 | CHUK | EGFR |
|  |  |  |  |  | FGF2 | NKX3-1 | Il6 | CSF1R | FGF7 | JUP |
|  |  |  |  |  | WNT5A | RAC1 | RHOA | NFKB1 | MLH1 |  |
|  |  |  |  |  | GSK3B | STAT3 | E2F1 | CDKN2A | KRAS | Hif1a |
| **p53 signaling pathway (hsa04115)** | 2 | miR-483-3p  miR-193-5p | 2 | 0.005609518 | TP73 | BBC3 |  |  |  |  |
| **PI3K-Akt signaling pathway hsa 04151:** | 2 | miR-155-5p  miR-148-5p | 26 | 0.01862909 | GSK3B | NFKB1 | CDK4 | THBS1 | MCL1 | CDK2 |
|  |  |  |  |  | RHEB | ITGB5 | COL4A2 | PPP2R2A | FLT1 | PDK1 |
|  |  |  |  |  | PKN2 | CSF1R | FGF7 | GNB4 | KRAS | FOXO3 |
|  |  |  |  |  | PCK2 | Csf1r | ITGB4 | EGFR | EIF4E2 | FGF2 |
|  |  |  |  |  | YWHAZ | RAC1 |  |  |  |  |
