# Supplementary material for: Conditioned medium of primary lung cancer cells induces EMT in A549 lung cancer cell line by TGF-ß1 and miRNA21 cooperation
Source: PLoS One. 2019 Jul 25;14(7):e0219597. doi: 10.1371/journal.pone.0219597 (PMC6657837; doi:10.1371/journal.pone.0219597)
Supplement: S2 Table — miRNA target genes expressed by the cells or released in the supernatants of LC212 and LC31 cell lines. (DOCX) [file pone.0219597.s002.docx]

**Supplementary material 2. Gene Ontology of miRNA target genes, specifically expressed by the cells or released in the supernatants of LC212 and LC31 cell lines.**

**GO analysis on the target genes of miRNAs increased in LC212 supernatants**

| **GOterm** | **Count** | **PValue** | **Genes** | | | | | | | | | | | | |
| --- | --- | --- | --- | --- | --- | --- | --- | --- | --- | --- | --- | --- | --- | --- | --- |
| **GO:0009719~response to endogenous stimulus** | 25 | 1.57E-16 | PLD1 | IL6 | MSH2 | TGFBR1 | ERBB2 | TGFBR2 | FOXO1 | RPS6KB1 | IL6R | APPL1 | PTEN | CCNA2 | PIK3R1 |
|  |  |  | BRCA1 | STAT3 | TGFB2 | CCNE1 | CDKN1A | CCND1 | EP300 | TSC1 | BCL2 | GNB4 | FAS | THBS1 |  |
| **GO:0042325~regulation of phosphorylation** | 23 | 3.98E-13 | EGFR | IL6 | PFKFB2 | TGFBR1 | ERBB2 | TGFBR2 | TTK | TLR4 | IL6R | CDK7 | CCNG1 | PTEN | CDC25A |
|  |  |  | TGFB2 | CCND1 | CDKN1A | CDKN1B | CDKN2B | TSC1 | BCL2 | PDGFD | THBS1 | APC |  |  |  |
| **GO:0051726~regulation of cell cycle** | 26 | 9.87E-20 | E2F1 | EGFR | E2F2 | MSH2 | SKP2 | TP53 | TTK | CDC23 | CDK6 | CDK7 | CDK4 | CCNG1 | PTEN |
|  |  |  | BRCA1 | CDC25A | CDK2 | TGFB2 | CCND1 | CDKN1A | CDKN1B | CDKN2B | BCL2 | SMC1A | MYC | CCNA2 | APC |
| **GO:0042981~regulation of apoptosis** | 30 | 3.37E-14 | TRAF1 | BID | MMP9 | ERBB2 | MITF | FOXO1 | TLR4 | PTEN | TGFB2 | BCL2 | FAS | THBS1 | MYC |
|  |  |  | APC | EGFR | MSH6 | IL6 | SGK3 | MSH2 | TGFBR1 | TP53 | SKP2 | IL6R | BIRC3 | BRCA1 | NRAS |
|  |  |  | CDKN1A | CDKN1B | VEGFA | APAF1 |  |  |  |  |  |  |  |  |  |
| **GO:0012501~programmed cell death** | 21 | 3.62E-09 | E2F1 | TRAF1 | BID | E2F2 | IL6 | LTBR | MSH2 | EGLN3 | TP53 | BIRC3 | PTEN | BRCA1 | NRAS |
|  |  |  | EP300 | CDKN1B | BCL2 | APAF1 | FAS | MDM4 | THBS1 | MYC |  |  |  |  |  |
| **GO:0005654~nucleoplasm** | 24 | 4.15E-10 | E2F1 | E2F2 | DBF4 | TP53 | CDC23 | RPS6KB2 | FOXO1 | CDK7 | NFKB2 | CDK4 | APPL1 | MCM5 | BRCA1 |
|  |  |  | CDK2 | CDC25A | CCNE1 | CCND1 | CDKN1A | EP300 | HIF1A | HOXA9 | TCF4 | MYC | CCNA2 |  |  |
| **GO:0051270~regulation of cell motion** | 16 | 2.99E-12 | EGFR | IL6 | PLD1 | MMP9 | TGFBR1 | RPS6KB1 | IL6R | PTEN | TGFB2 | HIF1A | CDKN1B | BCL2 | VEGFA |
|  |  |  | THBS1 | PIK3R1 | APC |  |  |  |  |  |  |  |  |  |  |
| **GO:0030334~regulation of cell migration** | 14 | 1.08E-10 | EGFR | PLD1 | IL6 | HIF1A | MMP9 | BCL2 | VEGFA | RPS6KB1 | IL6R | THBS1 | PTEN | PIK3R1 | TGFB2 |
|  |  |  | APC |  |  |  |  |  |  |  |  |  |  |  |  |
| **GO:0010604~positive regulation of macromolecule metabolic process** | 31 | 2.35E-14 | THBS1 | MYC | CCNA2 | APC | IL6 | TGFBR1 | TP53 | CDC23 | CDK7 | IL6R | CDK4 | BRCA1 | CDK2 |
|  |  |  | E2F1 | MITF | TTK | FOXO1 | TLR4 | TCF7L1 | TGFB2 | ARNT | CCNE1 | WNT1 | REL | BCL2 | TCF4 |
|  |  |  | STAT3 | CCND1 | HIF1A | EP300 | VEGFA |  |  |  |  |  |  |  |  |
| **GO:0022403~cell cycle phase** | 23 | 3.61E-14 | CCNE1 | CCND1 | CDKN1A | CDKN1B | CDKN2B | BCL2 | SMC1A | CCNA2 | STAG2 |  |  |  |  |
|  |  |  | E2F1 | EGFR | MSH6 | DBF4 | SKP2 | CDC23 | TTK | CDK6 | CDK4 | CCNG1 | HMGA2 | CDC25A | CDK2 |
| **GO:0070482~response to oxygen levels** | 10 | 4.69E-07 | CDKN1A | HIF1A | EP300 | BCL2 | TGFBR1 | VEGFA | EGLN3 | THBS1 | ARNT | TGFB2 |  |  |  |
| **GO:0001952~regulation of cell-matrix adhesion** | 6 | 9.35E-07 | TSC1 | BCL2 | CDK6 | THBS1 | PTEN | PIK3R1 |  |  |  |  |  |  |  |
| **GO:0010810~regulation of cell-substrate adhesion** | 6 | 1.43E-05 | TSC1 | BCL2 | CDK6 | THBS1 | PTEN | PIK3R1 |  |  |  |  |  |  |  |
| **GO:0001953~negative regulation of cell-matrix adhesion** | 3 | 0.001987 | THBS1 | PTEN | PIK3R1 |  |  |  |  |  |  |  |  |  |  |
| **GO:0010812~negative regulation of cell-substrate adhesion** | 3 | 0.0024178 | THBS1 | PTEN | PIK3R1 |  |  |  |  |  |  |  |  |  |  |
| **GO:0007162~negative regulation of cell adhesion** | 3 | 0.0344929 | THBS1 | PTEN | PIK3R1 |  |  |  |  |  |  |  |  |  |  |
| **GO:0007346~regulation of mitotic cell cycle** | 11 | 7.81E-08 | EGFR | CCND1 | CDKN2B | BCL2 | TP53 | CDC23 | TTK | CCNA2 | MYC | CDK2 | APC |  |  |
| **GO:0030856~regulation of epithelial cell differentiation** | 4 | 9.79E-04 | CCND1 | CDKN2B | TGFBR1 | APC |  |  |  |  |  |  |  |  |  |
| **GO:0030155~regulation of cell adhesion** | 9 | 4.10E-06 | TSC1 | ERBB2 | BCL2 | CDK6 | THBS1 | PTEN | PIK3R1 | TGFB2 | APC |  |  |  |  |
| **GO:0001952~regulation of cell-matrix adhesion** | 6 | 9.35E-07 | TSC1 | BCL2 | CDK6 | THBS1 | PTEN | PIK3R1 |  |  |  |  |  |  |  |
| **GO:0030949~positive regulation of vascular endothelial growth factor receptor signaling pathway** | 3 | 9.40E-04 | HIF1A | VEGFA | ARNT |  |  |  |  |  |  |  |  |  |  |
| **GO:0032844~regulation of homeostatic process** | 6 | 0.0010684 | HIF1A | BCL2 | VEGFA | CDK6 | MYC | ARNT |  |  |  |  |  |  |  |

**GO analysis on the target genes of miRNAs increased in LC212 cells**

| **GOterm** | **Count** | **PValue** | **Genes** | | | | | | | | | | | | |
| --- | --- | --- | --- | --- | --- | --- | --- | --- | --- | --- | --- | --- | --- | --- | --- |
| **GO:0010033~response to organic substance** | 26 | 8.83E-16 | MCL1 | ERBB2 | PFKFB2 | TLR4 | PTEN | TGFB2 | CCNE1 | BCL2 | FAS | THBS1 | MYC | PIK3R1 | EGFR |
|  |  |  | PLD1 | MSH2 | TGFBR1 | TGFBR2 | APPL1 | BRCA1 | STAT3 | CDKN1A | CCND1 | EP300 | TSC1 | GNB4 | IL6 |
| **GO:0042325~regulation of phosphorylation** | 22 | 1.81E-15 | EGFR | IL6 | PFKFB2 | ERBB2 | TGFBR1 | TGFBR2 | TTK | CHEK1 | TLR4 | CCNG1 | PTEN | CDC25A | TGFB2 |
|  |  |  | CDC42 | CCND1 | CDKN1A | TSC1 | BCL2 | PDGFD | THBS1 | APC | CCNE2 |  |  |  |  |
| **GO:0042981~regulation of apoptosis** | 24 | 9.77E-13 | EGFR | BID | MSH6 | IL6 | SGK3 | MCL1 | MSH2 | MMP9 | ERBB2 | TGFBR1 | TP53 | SKP2 | TLR4 |
|  |  |  | BRCA1 | TGFB2 | CDKN1A | BCL2 | VEGFA | APAF1 | FAS | THBS1 | MYC | APC | PTEN |  |  |
| **GO:0051272~positive regulation of cell motion** | 11 | 3.21E-11 | EGFR | PLD1 | IL6 | MMP9 | BCL2 | TGFBR1 | VEGFA | THBS1 | PIK3R1 | TGFB2 | APC |  |  |
| **GO:0007049~cell cycle** | 31 | 1.38E-20 | E2F2 | CDC14A | TTK | CHEK1 | CCNG1 | SESN1 | TGFB2 | CCNE2 | CCNE1 | BCL2 | THBS1 | MYC | STAG2 |
|  |  |  | APC | EGFR | MSH6 | MSH2 | TP53 | SKP2 | CDK6 | CDK4 | APPL1 | BRCA1 | CDC25A | WEE1 | CDKN1A |
|  |  |  | E2F1 | CCND1 | EP300 | MDM4 | SMC1A |  |  |  |  |  |  |  |  |
| **GO:0006915~apoptosis** | 16 | 1.17E-07 | E2F1 | BID | E2F2 | IL6 | MCL1 | MSH2 | TP53 | PTEN | BRCA1 | EP300 | BCL2 | APAF1 | MDM4 |
|  |  |  | FAS | THBS1 | MYC |  |  |  |  |  |  |  |  |  |  |
| **GO:0006928~cell motion** | 15 | 4.22E-08 | IL6 | ERBB2 | PTEN | STAT3 | TGFB2 | VCL | WNT1 | PTK2 | PVRL1 | SERPINB5 | THBS1 | APC | FN1 |
|  |  |  |  | MSH2 | TGFBR1 |  |  |  |  |  |  |  |  |  |  |
| **GO:0051247~positive regulation of protein metabolic process** | 11 | 2.19E-07 | CCND1 | IL6 | EP300 | BCL2 | TGFBR1 | TTK | TLR4 | CDK4 | THBS1 | BRCA1 | APC |  |  |
| **GO:0006796~phosphate metabolic process** | 22 | 2.30E-09 | CDC25A | WEE1 | TGFB2 | PTK2 | CCND1 | BCL2 | THBS1 | CHUK | PIK3R1 |  |  |  |  |
|  |  |  | EGFR | CDC14A | SGK3 | MSH2 | ERBB2 | TGFBR1 | TGFBR2 | MKNK2 | TTK | CDK6 | CHEK1 | CDK4 | PTEN |
| **GO:0001952~regulation of cell-matrix adhesion** | 6 | 1.63E-07 | TSC1 | BCL2 | CDK6 | THBS1 | PTEN | PIK3R1 |  |  |  |  |  |  |  |
| **GO:0008285~negative regulation of cell proliferation** | 15 | 1.28E-09 | TGFBR2 | TP53 | CDK6 | CHEK1 | PTEN | SESN1 | TGFB2 | CDKN1A | TSC1 | BCL2 | MDM4 | THBS1 | APC |
|  |  |  | IL6 | ERBB2 |  |  |  |  |  |  |  |  |  |  |  |
| **GO:0030155~regulation of cell adhesion** | 9 | 2.80E-07 | TSC1 | ERBB2 | BCL2 | CDK6 | THBS1 | PTEN | PIK3R1 | TGFB2 | APC |  |  |  |  |
| **GO:0050678~regulation of epithelial cell proliferation** | 6 | 2.23E-05 | EGFR | ERBB2 | VEGFA | CDK6 | TGFB2 | APC |  |  |  |  |  |  |  |
| **GO:0042493~response to drug** | 12 | 5.61E-09 | CCNE1 | CDKN1A | CCND1 | EP300 | MSH2 | ERBB2 | BCL2 | TGFBR2 | TP53 | PTEN | STAT3 | APC |  |
| **GO:0001952~regulation of cell-matrix adhesion** | 6 | 1.63E-07 | TSC1 | BCL2 | CDK6 | THBS1 | PTEN | PIK3R1 |  |  |  |  |  |  |  |
| **GO:0043062~extracellular structure organization** | 7 | 1.28E-04 | PTK2 | TSC1 | MMP9 | ERBB2 | TGFBR1 | COL5A2 | TGFB2 |  |  |  |  |  |  |

**GO analysis on the target genes of miRNAs increased in LC31 supernatants**

| **GOTerm** | **Count** | **PValue** | **Genes** | | | | | | | | | | | | |
| --- | --- | --- | --- | --- | --- | --- | --- | --- | --- | --- | --- | --- | --- | --- | --- |
| **GO:0043067~regulation of programmed cell death** | 21 | 2.00E-11 | BBC3 | ETS1 | GSK3B | RAC1 | RHOA | THBS1 | FGF2 | APC | NFKB1 | FOXO3 | TP73 | CDKN2A | KRAS |
|  |  |  | EGFR | MSH6 | YWHAZ | IL6 | MCL1 | MSH2 | MLH1 | SMAD3 |  |  |  |  |  |
| **GO:0051094~positive regulation of developmental process** | 15 | 3.54E-12 | FGF2 | APC | SMAD4 | SMAD3 | NFKB1 | SMAD2 | FOXO3 | SMAD1 | CTNNB1 | HIF1A | ETS1 | RHOA | THBS1 |
|  |  |  | IL6 | SMAD5 |  |  |  |  |  |  |  |  |  |  |  |
| **GO:0042981~regulation of apoptosis** | 20 | 1.52E-10 | EGFR | BBC3 | ETS1 | GSK3B | RAC1 | RHOA | THBS1 | APC | NFKB1 | FOXO3 | TP73 | CDKN2A | KRAS |
|  |  |  | MSH6 | YWHAZ | IL6 | MCL1 | MSH2 | MLH1 | SMAD3 |  |  |  |  |  |  |
| **GO:0032583~regulation of gene-specific transcription** | 8 | 1.09E-06 | IL6 | HIF1A | ETS1 | SPI1 | SMAD3 | NFKB1 | SMAD2 | CTNNB1 |  |  |  |  |  |
| **GO:0006796~phosphate metabolic process** | 16 | 4.97E-06 | EGFR | MSH2 | PKN2 | SMAD2 | CDK4 | CDK2 | GSK3B | YES1 | THBS1 | FGF2 | CHUK | CSF1R | PPP2R2A |
|  |  |  | PTPRJ | PDK1 | FLT1 |  |  |  |  |  |  |  |  |  |  |
| **GO:0051130~positive regulation of cellular component organization** | 8 | 8.07E-06 | GSK3B | RAC1 | RHOA | SMAD4 | SMAD3 | SMAD2 | SMAD1 | APC |  |  |  |  |  |
| **GO:0042127~regulation of cell proliferation** | 19 | 9.01E-10 | EGFR | SMAD3 | SMAD2 | SMAD1 | CDK4 | CDK2 | CTNNB1 | CDKN2A | HIF1A | KRAS | ETS1 | NKX3-1 | THBS1 |
|  |  |  | IL6 | FGF7 | FLT1 | SMAD4 | FGF2 | APC |  |  |  |  |  |  |  |
| **GO:0051272~positive regulation of cell motion** | 9 | 5.22E-09 | EGFR | IL6 | FLT1 | HIF1A | ETS1 | SMAD3 | THBS1 | FGF2 | APC |  |  |  |  |
| **GO:0030335~positive regulation of cell migration** | 8 | 6.59E-08 | EGFR | IL6 | FLT1 | HIF1A | SMAD3 | THBS1 | FGF2 | APC |  |  |  |  |  |

**GO analysis on the target genes of miRNAs incresead in LC31 cells**

| **Term** | **Count** | **PValue** | **Genes** | | | | | | | | | | | | |
| --- | --- | --- | --- | --- | --- | --- | --- | --- | --- | --- | --- | --- | --- | --- | --- |
| **GO:0043067~regulation of programmed cell death** | 25 | 1.57E-14 | APC | EGFR | MSH6 | MSH2 | SMAD3 | IL6R | CDKN1A | BBC3 | ETS1 | BAX | GSK3B | VEGFA | FGF2 |
|  |  |  | YWHAZ | MCL1 | MLH1 | PRKDC | NFKB1 | FOXO3 | CDKN2A | KRAS | RAC1 | RHOA | FAS | THBS1 |  |
| **GO:0051094~positive regulation of developmental process** | 16 | 7.85E-13 | SMAD5 | SMAD4 | SMAD3 | NFKB1 | SMAD2 | IL6R | FOXO3 | SMAD1 | CTNNB1 | HIF1A | ETS1 | BAX | RHOA |
|  |  |  | THBS1 | FGF2 | APC |  |  |  |  |  |  |  |  |  |  |
| **GO:0022402~cell cycle process** | 19 | 2.06E-11 | EGFR | MSH6 | MSH2 | MLH1 | SMAD3 | CDK6 | CDK4 | WEE1 | CDC25A | CDK2 | CTNNB1 | CDKN1A | CDKN2A |
|  |  |  | PLK1 | GSK3B | MDM2 | THBS1 | STAG2 | APC |  |  |  |  |  |  |  |
| **GO:0010033~response to organic substance** | 16 | 3.87E-07 | EGFR | MCL1 | MSH2 | SMAD3 | PRKDC | SMAD2 | IL6R | SMAD1 | STAT3 | CTNNB1 | CDKN1A | KRAS | GNB4 |
|  |  |  | FAS | THBS1 | PPP2R2A |  |  |  |  |  |  |  |  |  |  |
| **GO:0051272~positive regulation of cell motion** | 10 | 4.12E-10 | EGFR | FLT1 | HIF1A | ETS1 | VEGFA | SMAD3 | IL6R | THBS1 | FGF2 | APC |  |  |  |
| **GO:0032268~regulation of cellular protein metabolic process** | 13 | 9.03E-07 | EGFR | SMAD4 | NFKB1 | IL6R | CDK4 | CDKN2A | PLK1 | BAX | MDM2 | PAK1 | THBS1 | FGF2 | EIF4E2 |
| **GO:0051130~positive regulation of cellular component organization** | 8 | 1.45E-05 | GSK3B | RAC1 | RHOA | SMAD4 | SMAD3 | SMAD2 | SMAD1 | APC |  |  |  |  |  |
