# Supplementary figures and images for: Conditioned medium of primary lung cancer cells induces EMT in A549 lung cancer cell line by TGF-ß1 and miRNA21 cooperation
Source: PLoS One. 2019 Jul 25;14(7):e0219597. doi: 10.1371/journal.pone.0219597 (PMC6657837; doi:10.1371/journal.pone.0219597)

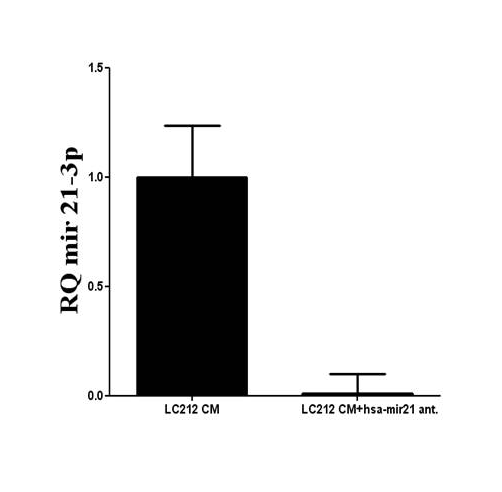

Supplement: S1 Fig — Relative quantification of hsa miR-21-3p relased in LC212-CM and in LC212-CM treated with hsa-miR-21 antagomir. (TIF) [file pone.0219597.s003.tif]

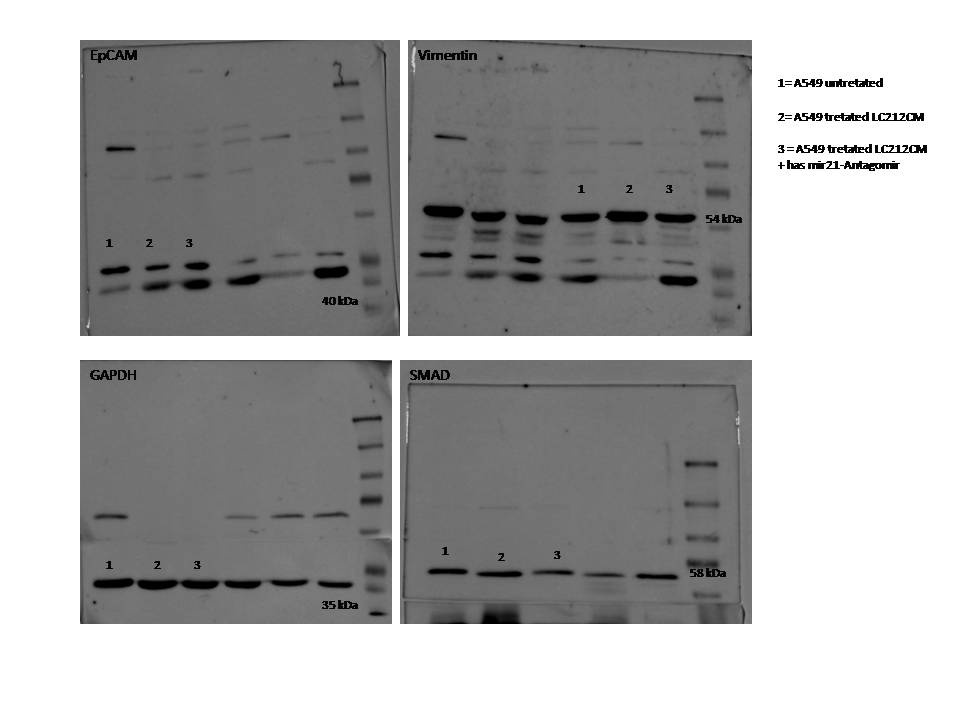

Supplement: S2 Fig — (TIF) [file pone.0219597.s004.tif]

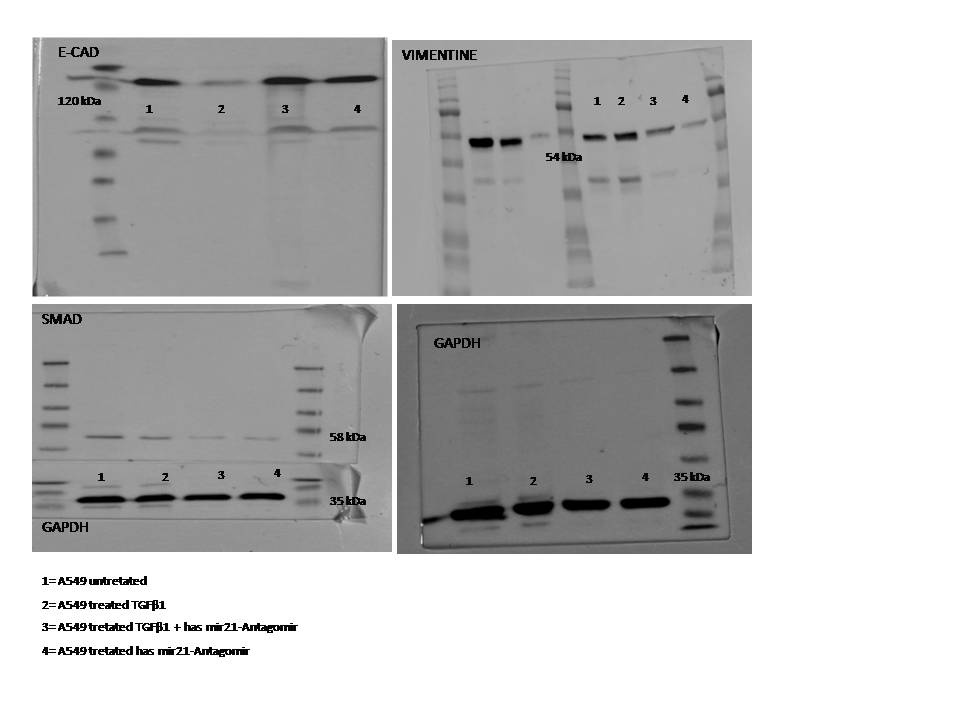

Supplement: S3 Fig — (TIF) [file pone.0219597.s005.tif]

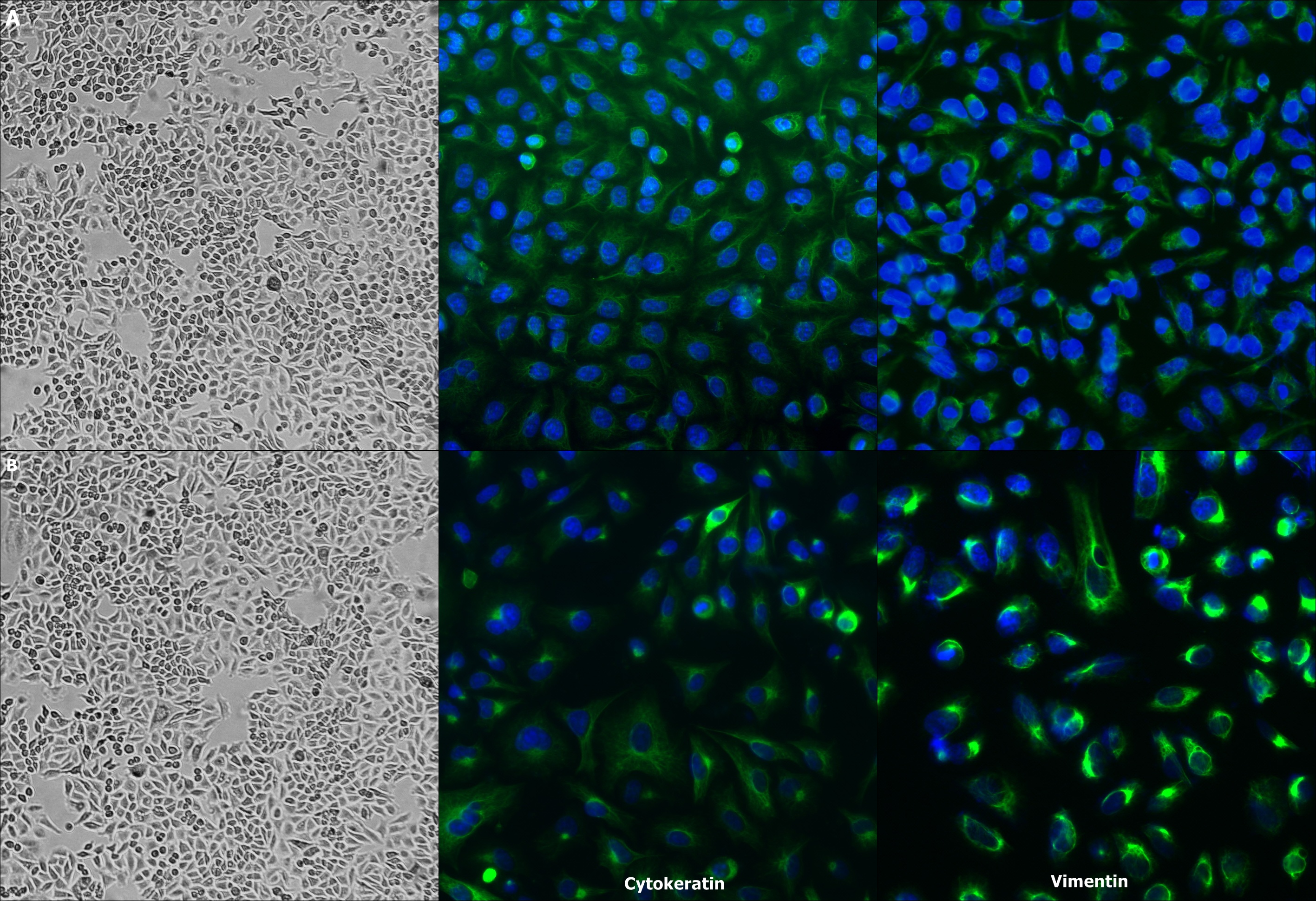

Supplement: S4 Fig — (A)A549 cells untreated (B) A549 cells conserved the epithelial morphology after treatment of conditioned medium derived to LC31 cell line for 48h. Morfological and Immunofluorescence assay. (TIF) [file pone.0219597.s006.tif]

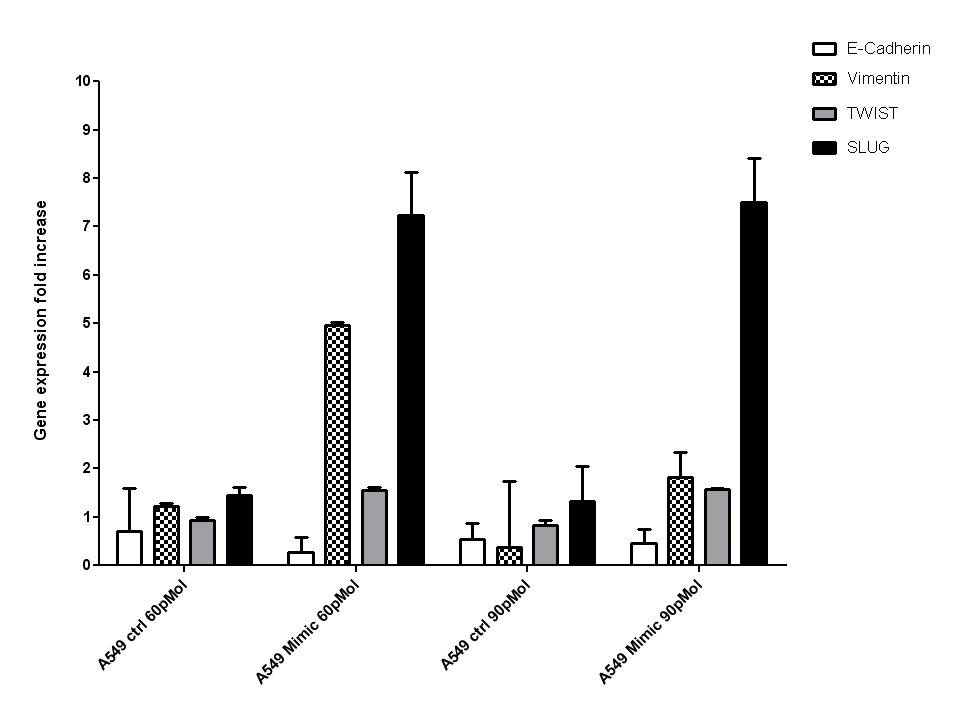

Supplement: S5 Fig — Relative quantification of EMT markes showed the over-expression of Vimentin, SLUG and TWIST and downregulation of E-Cadherin after transfection with hsa-mir-21-3p Mimic (used 60 pMol and 90 pMol for 48h) on A549 cells. (TIF) [file pone.0219597.s007.tif]
